# Spectroscopic and Rheological Characterization of Polyvinyl Alcohol/Hyaluronic Acid-Based Systems: Effect of Polymer Ratio and Riboflavin on Hydrogel Properties

**DOI:** 10.3390/gels11100773

**Published:** 2025-09-25

**Authors:** Iulia Matei, Marius Alexandru Mihai, Sorina-Alexandra Leau, Ludmila Aricov, Anca Ruxandra Leonties, Elvira Alexandrescu, Gabriela Ionita

**Affiliations:** 1 “Ilie Murgulescu” Institute of Physical Chemistry of the Romanian Academy, 202 Splaiul Independentei, 060021 Bucharest, Romania; iuliamatei@icf.ro (I.M.); amihai@icf.ro (M.A.M.); sleau@icf.ro (S.-A.L.); laricov@icf.ro (L.A.); aleonties@icf.ro (A.R.L.); 2 Department of Analytical Chemistry and Environmental Engineering, Faculty of Chemical Engineering and Biotechnologies, National University of Science and Technology Politehnica of Bucharest, 1-7 Polizu Gheorghe, 011061 Bucharest, Romania; 3 National Institute for Research & Development in Chemistry and Petrochemistry—ICECHIM Bucharest, 202 Splaiul Independentei, 060021 Bucharest, Romania; elvira.alexandrescu@icechim.ro

**Keywords:** EPR spectroscopy, hydrogels, polyvinyl alcohol, hyaluronic acid, 4-amino-TEMPO, riboflavin

## Abstract

We report a systematic investigation on the physicochemical properties of polymer systems consisting of polyvinyl alcohol (PVA) and hyaluronic acid (HA) mixed in various volume ratios (1/4, 2/3, 1/1, 3/2, and 4/1). At PVA/HA ratios above 1/1, in the presence of glutaraldehyde and divinyl sulfone as crosslinking agents, hydrogels are formed. Their swelling behavior is dependent on the polymer ratio, with the highest water uptake determined for PVA/HA 4/1. The in situ generation of reactive oxygen species (HO^•^ radicals) under UV-A irradiation, in the presence of riboflavin as a photoinitiator, is evidenced by electron paramagnetic resonance (EPR) spectroscopy. The diffusion of small paramagnetic molecules across the interface of two PVA/HA 4/1 gel pieces placed in direct contact reveals the occurrence of molecular exchange, which could indicate some degree of self-repair of the hydrogel network. When the paramagnetic moiety is attached to the HA polymer by spin labeling, the absence of diffusion demonstrates the stability of the crosslinked HA chains within the PVA/HA network. The structural modifications induced by crosslinking, by the presence of riboflavin, and by exposure to UV-A light, and the resulting alterations in the mechanical behavior of the hydrogels are monitored by infrared spectroscopy and rheology. Only a slight decrease in the viscoelastic moduli values is noted, indicating that the formation of HO^•^ radicals has minimal impact on the macroscopic properties of the hydrogels.

## 1. Introduction

The three-dimensional network of hydrogels is the resultant of chemical or physical crosslinking of water-soluble polymers that can accommodate different amounts of water depending on the network density. A particular case of hydrogel is represented by the interpenetrating polymer networks (IPNs) formed when one polymer network is generated in the presence of another, resulting in a more adaptable material that displays a combination of the properties of the constituting partners. As a class of soft biomaterials, such hydrogels find diverse applications in the biomedical field, notably for drug delivery and tissue engineering [1,2,3,4].

**Scheme 1:**
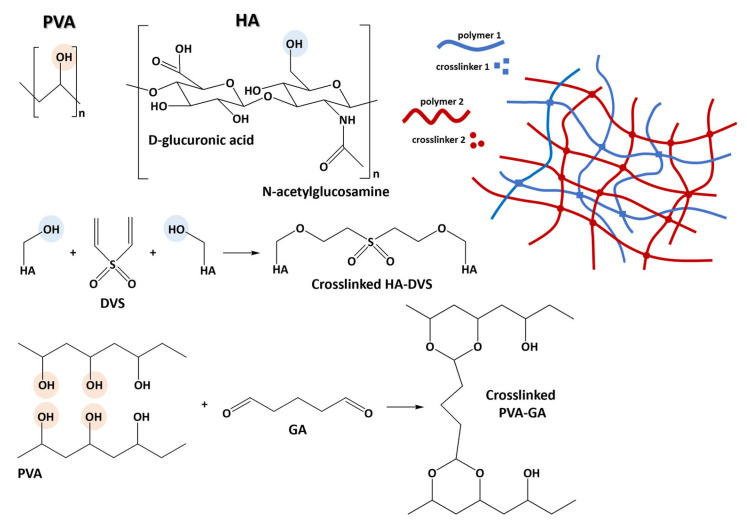
Chemical structures of polyvinyl alcohol (PVA) and hyaluronic acid (HA), crosslinking reactions of PVA with glutaraldehyde (GA, via acetal bridges) and of HA with divinyl sulfone (DVS, via ether bonds), and schematic representation of the resulting gel network.

In this study, we investigate IPN networks formed by polyvinyl alcohol (PVA) and hyaluronic acid (HA) in the presence of their respective crosslinking agents, glutaraldehyde (GA) and divinyl sulfone (DVS). The crosslinking reactions and structure of the resulting gel network are depicted in Scheme 1. The choice of these two polymers is based on their distinctive properties: PVA is biocompatible and can generate hydrogels for applications as artificial soft lenses [5,6], while HA is a component of the extracellular matrix and can be found as an active ingredient in pharmaceuticals like artificial tear drops used in the treatment of dry eye syndrome [7]. In addition to their relevance for ophthalmic applications, the PVA and HA polymers show mechanical properties like strength [8] and elasticity [9], respectively, that make them ideal IPN building blocks. PVA/HA crosslinking can prevent the dissolution of PVA in water and stabilize HA, whose biomedical applications are otherwise limited by degradation [10]. It is noteworthy to mention that hydrogels based on either PVA or HA possess the ability to encapsulate various (macro)molecules [11].

Our study reports on the physicochemical properties (swelling behavior, mechanical properties) of PVA/HA materials with variable composition, on the diffusion processes of encapsulated small molecules, and on the possibility to generate reactive oxygen species (ROS) in these systems under UV-A irradiation. The investigation combines gravimetric analysis, rheology, infrared spectroscopy, and electron paramagnetic resonance (EPR) spectroscopy. Two small molecules have been selected: the photoinitiator riboflavin and the paramagnetic probe 4-amino-TEMPO (4-amino-2,2,6,6-tetramethyl-1-piperidinyloxy). Riboflavin is routinely used in the treatment of ocular diseases due to its capacity to generate ROS under UV-A light and initiate collagen crosslinking [12]. Using EPR spectroscopy, we have previously shown that riboflavin determines the formation in collagen of regular interfibrillary bonds, a process that increases the corneal mechanical strength [13]. The EPR technique is also suitable for monitoring eye disease progression and treatment by following the protein profile of tear samples [14]. In a series of studies, we employed EPR spectroscopy to collect information at the molecular level on various systems with supramolecular organization, including gels. For instance, the spin labeling method was used to evidence the formation of alginate/chitosan IPN networks [15], while spin probes with various molecular weights were used to evaluate diffusional properties in covalent, ionotropic, semi-IPN, and IPN hydrogels [16]. The latter study constitutes the starting point for our present investigation. Here, we aim to demonstrate that EPR spectroscopy also represents a convenient tool to monitor processes that occur at the nanoscopic level in PVA/HA hydrogels. By spin labeling the HA polymer with 4-amino-TEMPO prior to crosslinking with PVA, we were able to prove the stability of the HA hydrogel network within the IPN structure. By using 4-amino-TEMPO as a spin probe, we evidenced the diffusion across the interface of two PVA/HA 4/1 gel pieces placed in direct contact, which indicates the occurrence of molecular exchange and some degree of self-repair of the hydrogel network. Conventionally, such processes are investigated by methods like rheology that only offer information at a global scale, as opposed to the local information provided by EPR.

## 2. Results and Discussion

The PVA/HA samples were prepared by mixing solutions of 5% PVA and 1% HA in various PVA/HA volume ratios (1/0; 4/1; 3/2; 1/1; 2/3; 1/4; 0/1), as described in the Materials and Methods section. The sol-gel transition behavior of these systems was observed by the tube inversion method [10] at 1 h and at 24 h from mixing the solutions of polymer(s) and crosslinker(s) (Figure S1 in Supplementary Materials). One observes that, at 1 h from sample preparation, the PVA/HA systems appear, depending on the PVA/HA ratio, either as viscous solutions (0/1, 1/4, 2/3) or as gels (1/1, 3/2, 4/1, 1/0). After 24 h from preparation, all PVA/HA systems except 0/1 and 1/4 are found in the gel phase.

### 2.1. Swelling Properties of PVA/HA Hydrogels

**Figure 1:**
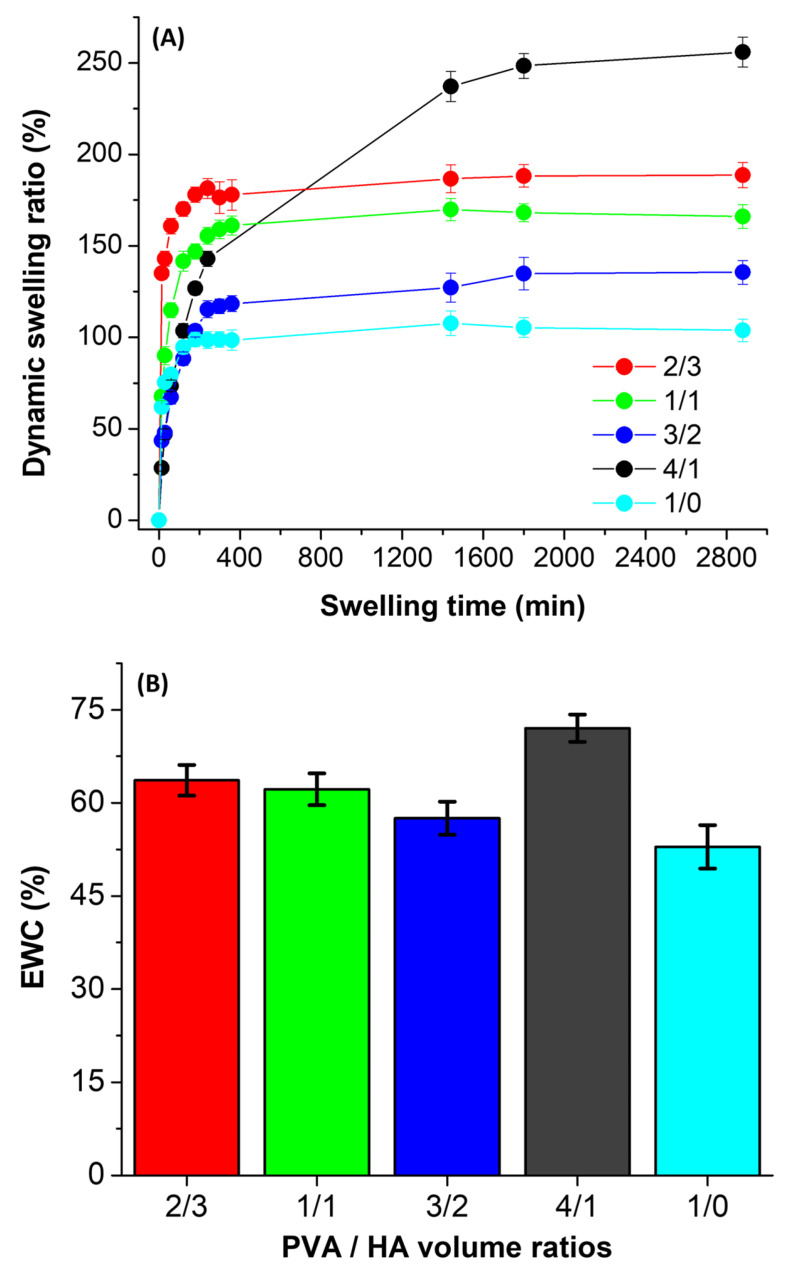
Swelling behavior of PVA/HA hydrogels in water revealed by the dynamic swelling ratio (**A**) and the equilibrium water content (**B**).

The initial water content of the freshly prepared PVA/HA gels was calculated as the ratio between the weight loss upon drying and the initial weight of the hydrated gel, and the values are listed in Table 1. The swelling behavior of the dry gels was then characterized by measuring the weight gain upon immersion in water. In Figure 1A is presented the variation over time of the dynamic swelling ratio, calculated using Equation (1). The equilibrium swelling ratio values, determined after an immersion time of 5 days (Figure S2), are given in Table 1. The corresponding equilibrium water content (EWC) values in Equation (2) are presented in Figure 1B. We observe that the PVA/HA hydrogels show large swelling ratios and EWC values that increase in the order 1/0 < 3/2 < 1/1 < 2/3 < 4/1.

The swelling capacity and swelling rate of IPN hydrogels are determined by an interplay between the density of crosslinks and the hydrophilicity of the two polymer networks [17,18]. The large number of crosslinks formed by the less hydrophilic polymer (i.e., PVA) reduces the available space between polymer chains, resulting in a rigid hydrogel matrix less prone to expand to accommodate water (PVA/HA 1/0). Addition of a polymer bearing a significantly larger number of hydrophilic groups (i.e., HA) favors water uptake by its inherent hydrophilicity, as well as by disrupting the formation of PVA crosslinks and creating a more flexible matrix (PVA/HA 3/2 < 1/1 < 2/3). A too high HA content weakens the IPN network to the point of its collapse (PVA/HA 1/4). The case of the PVA/HA 4/1 hydrogel stands out. The highest swelling capacity observed at this composition seems to indicate an optimal balance between the properties of the two polymers. The substantial amount of PVA provides structural integrity, preventing against gel degradation and collapse, while the amount of HA is sufficient to significantly increase the hydrogel’s ability to absorb water.

Taking into consideration that the PVA/HA 4/1 hydrogel shows the best swelling capacity, we have focused our further investigations on analyzing its physicochemical properties in the absence and in the presence of spin probes and riboflavin. The ability of this hydrogel to hold large amounts of water is expected to facilitate the diffusion of small molecules within the hydrogel matrix and the generation and detection of ROS while providing a more biologically relevant environment in view of potential ophthalmic applications.

### 2.2. Diffusion of Low Molecular Weight Compounds in PVA/HA 4/1 Hydrogel and Gel Network Stability

**Figure 2:**
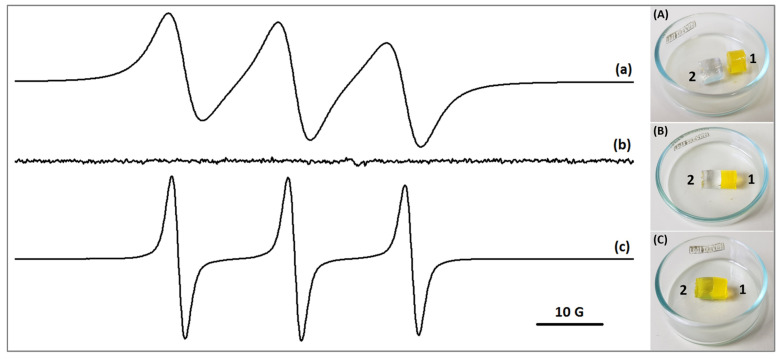
Left panel: The EPR spectra evidencing the diffusion of 4-amino-TEMPO in PVA/HA 4/1 hydrogel: (**a**) gel sample 1 impregnated with 4-amino-TEMPO, prior to diffusion; (**b**) blank gel sample 2 prior to diffusion; (**c**) gel sample 2 after diffusion of 4-amino-TEMPO from sample 1 (impregnated) to sample 2 (initially blank). Spectrum (**c**) was recorded 24 h after contact. Right panel: (**A**) Gel sample 1 (impregnated with riboflavin, yellow) and gel sample 2 (blank, colorless); (**B**) samples immediately after contact; (**C**) samples 24 h after contact.

The diffusional properties in PVA/HA 4/1 hydrogel were investigated using two molecular probes, riboflavin—as a yellow-colored dye and photoinitiator—and 4-amino-TEMPO—as a paramagnetic species. A PVA/HA 4/1 gel sample loaded with riboflavin and with 4-amino TEMPO (gel sample 1, yellow coloration in Figure 2, right panel) was placed in close contact with a blank PVA/HA 4/1 sample (gel sample 2, colorless). The diffusion of the two molecular probes from sample 1 (impregnated) to sample 2 (blank), through the contact surface, was evidenced both visually and by EPR spectroscopy (Figure 2, left panel). A yellow coloration of sample 2 was observed after 24 h, indicating diffusion of riboflavin across the interface. Similarly, the diffusion of 4-amino-TEMPO in the initially blank PVA/HA 4/1 sample (spectrum (b) in Figure 2, no EPR signal) was evidenced by recording an EPR signal of sample 2 after 24 h from contact (spectrum (c), the characteristic EPR signal of 4-amino-TEMPO). The line broadening observed in spectrum (a) for gel sample 1 is due to the high concentration of 4-amino-TEMPO entrapped within the solvent pools of the gel network. The appearance of an EPR signal in sample 2 demonstrates the occurrence of molecular exchange and points to some degree of self-repair of the hydrogel network.

The EPR spectroscopy, through its spin labeling method, is ideal to investigate whether polymeric chains are able to diffuse within the PVA/HA hydrogel matrix. For this purpose, HA was spin labeled with 4-amino-TEMPO prior to crosslinking (HA^•^). We then prepared a different gel sample 1’, by crosslinking PVA with HA^•^ in a 4/1 ratio. The PVA/HA^•^ 4/1 sample 1’, also impregnated with riboflavin, was placed in contact with a blank PVA/HA 4/1 sample 2’. While riboflavin diffusion through the contact surface was observed once again, no EPR signal was recorded from gel sample 2’ upon monitoring for 7 days. This indicates that the HA^•^ polymeric chains do not diffuse through the hydrogel matrix, demonstrating the effective crosslinking and immobilization of the HA network within the IPN structure.

### 2.3. Reactive Oxygen Species Generation in PVA/HA Hydrogel

**Figure 3:**
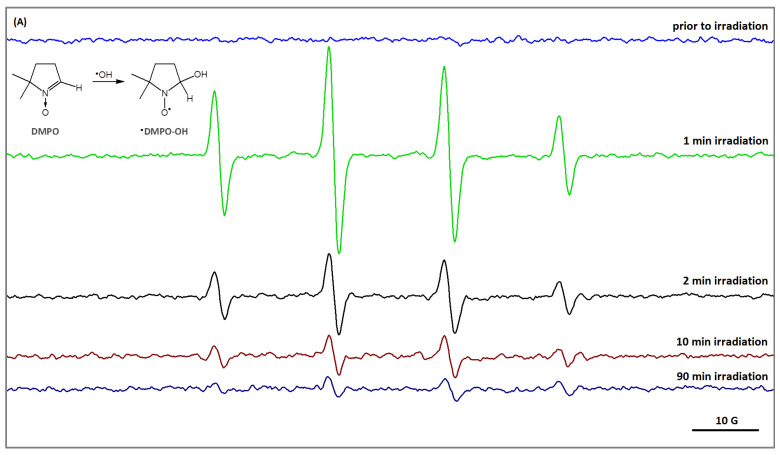

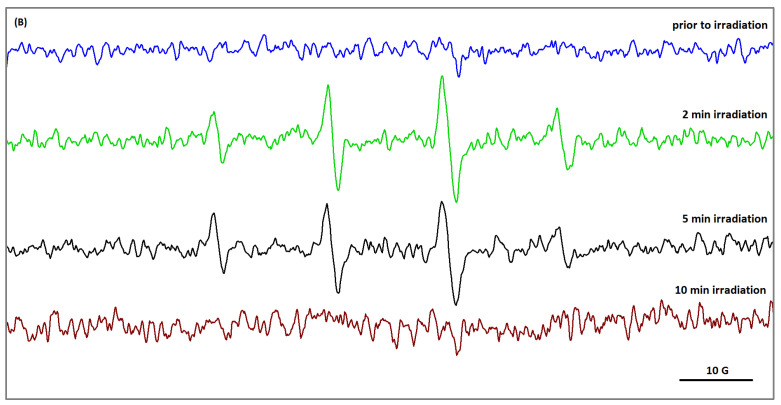
The EPR spectra of the PVA/HA 4/1 hydrogel in the presence (**A**) and in the absence (**B**) of encapsulated riboflavin, recorded under UV-A irradiation at different irradiation times.

The spin trapping method of EPR spectroscopy was used to test the generation, under UV-A irradiation, of free radicals in the PVA/HA 4/1 hydrogel, in the absence or presence of riboflavin in the gel matrix. The DMPO spin trap (5,5-dimethyl-1-pyrroline N-oxide) was selected for this purpose, knowing that it forms stable spin adducts with reactive oxygen and carbon species, thus providing characteristic EPR line patterns for each. In our case, a 1:2:2:1 quartet signal was recorded, characteristic of the ^•^DMPO-OH spin adduct (inset of Figure 3A). This evidences the generation of hydroxyl (HO^•^) radicals within the PVA/HA 4/1 hydrogel network. The values of the hyperfine splitting constants of the ^•^DMPO-OH adduct, obtained by spectrum simulation, are a_N_ = 14.95 G and a_H_^β^ = 14.76 G, in accordance with the values reported by Buettner [19]. The generation of HO^•^ radicals is notable in the presence of riboflavin (Figure 3A), although a weak signal corresponding to the ^•^DMPO-OH adduct is also observed in the absence of riboflavin (Figure 3B). The samples are exposed to air, so O_2_ under UV-A light can generate HO^•^ radicals inside the hydrogel. The intensity of the EPR signal of the ^•^DMPO-OH adduct generated in the PVA/HA/riboflavin sample decreases over time, suggesting that the radicals are consumed in further reactions, most probably due to the presence of HA [20].

Upon irradiation of the spin-labeled PVA/HA^•^ 4/1 hydrogel, the EPR signal of 4-amino-TEMPO disappears within 15 min for the PVA/HA^•^ sample and within 5 min for the PVA/HA^•^/riboflavin sample (Figure S3). Since nitroxides are known to act as scavengers of free radicals [21], the experimental observations can be explained by the consumption of 4-amino-TEMPO in reaction with the HO^•^ radicals generated by riboflavin and HA^•^. The faster decay rate of 4-amino-TEMPO in the PVA/HA^•^/riboflavin gel matrix can be correlated to the larger concentration of free radicals generated in this system.

The presence within the gel matrix of free radicals generated by riboflavin may have an effect on the structure of the gel network. Such structural alterations usually determine a change in the physicochemical properties of the gel. Therefore, in order to evidence the influence of riboflavin and the effect of HO^•^ radicals on the gel network, we recorded the infrared spectra and performed rheological measurements on these systems.

### 2.4. Infrared Spectroscopy of PVA/HA Hydrogels

**Figure 4:**
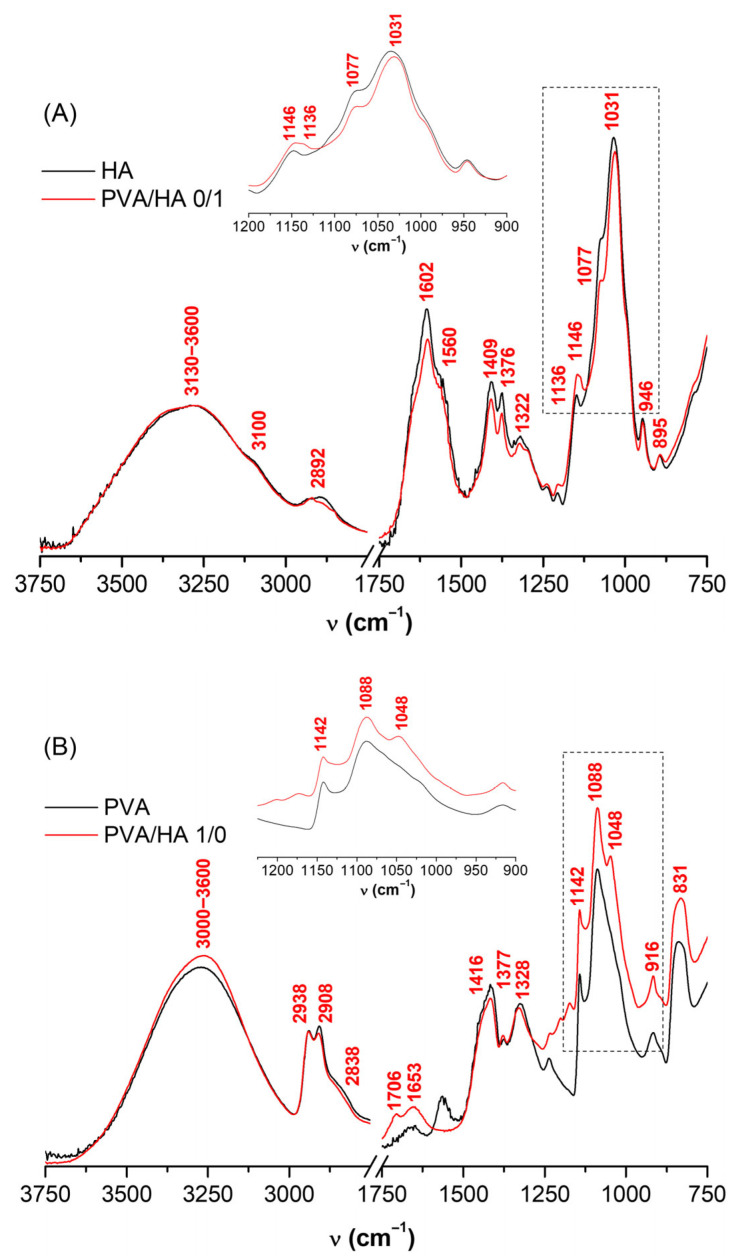

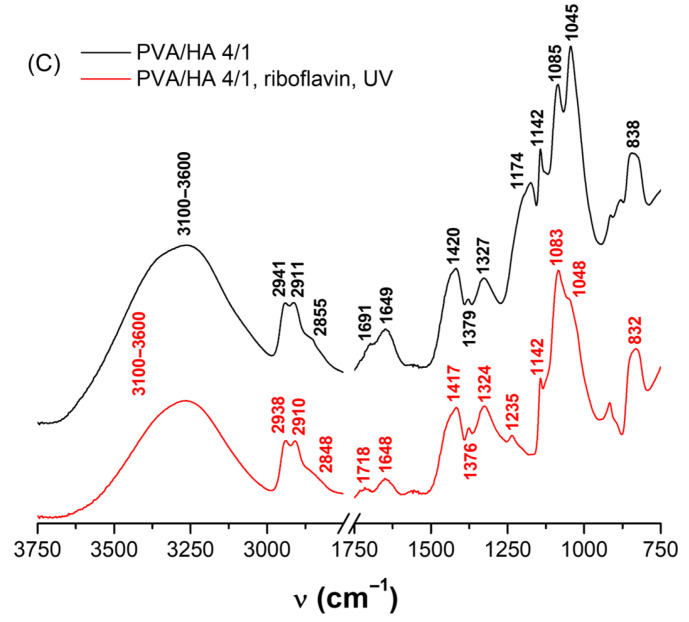
Infrared spectra of HA prior to and after crosslinking with DVS (**A**), of PVA prior to and after crosslinking with GA (**B**), and of the PVA/HA 4/1 system (**C**) in the absence of riboflavin (black) and in the presence of riboflavin, after UV-A irradiation (red). In (**A**,**B**), the spectral regions marked on the figure are presented as insets.

Infrared spectroscopy was used to evidence structural differences induced by crosslinking and by the presence of riboflavin under UV-A exposure. Spectral shifts, band broadenings, and changes in relative intensity provide insight into the crosslinking process. Infrared spectra of dried films of crosslinked PVA/HA 4/1, with or without loaded riboflavin, before or after UV-A irradiation, are shown in Figure 4, together with reference spectra of PVA crosslinked with GA (PVA/HA 1/0), HA crosslinked with DVS (PVA/HA 0/1), and pure PVA and HA. Characteristic vibrational bands have been assigned according to literature data [22,23,24,25], and this information is presented in Table S1 and discussed below.

In the case of HA, the infrared spectra recorded before and after crosslinking with DVS (Figure 4A) do not evidence striking changes, as previously noted by Tomihata and Ikada [26]. The most intense band of HA is centered at 1035 cm^−1^, with a shoulder at 1077 cm^−1^ and another less intense band at 1148 cm^−1^. These characteristic bands of HA are ascribed to ν_C-OH_ and to ν_C-O-C_ stretching vibrations, respectively [22,23]. Since C-H stretching remains virtually unchanged upon crosslinking [27], we considered the ν_C-H_ band at 2892 cm^−1^ as a control and computed the intensity ratio I(ν_C-O-C_)/I(ν_C-H_) prior to and after crosslinking with DVS. We observed an increase in this ratio from 2.4 in the HA sample to 2.9 in the PVA/HA 0/1 system, which evidences the increase in the number of C-O-C linkages upon crosslinking. The new band observed at 1136 cm^−1^ in the spectrum of the crosslinked sample corresponds to the S=O symmetric stretching vibration of the sulfone group.

The crosslinking of PVA by GA is evidenced in the infrared spectra (Figure 4B) by changes in the 1020–1230 cm^−1^ spectral region that comprises δ_O-H_ bending coupled with ν_C-OH_ stretching vibrations of PVA. Most notable is the change in the intensity ratio of the bands at 1088 and 1048 cm^−1^, the latter band appearing as a slight shoulder in pure PVA and as a sharp band in the crosslinked PVA/HA 1/0 sample. These changes reflect the rising contribution of C-O-C and O-C-O vibrations of the acetal ring formed by crosslinking [24]. The band at 1142 cm^−1^, corresponding to ν_C-C_ and ν_C-O_ vibrations in the carbon framework of the PVA chain, is correlated to the crystallinity of the sample and can be used to assess the latter. A sharp band at 1142 cm^−1^, as in our case, indicates a semi-crystalline polymer, while a shift of this band to 1154 cm^−1^ would indicate an amorphous structure [28]. The weak band at 1706 cm^−1^ is ascribed to C=O stretching vibrations in GA. Its presence indicates that not all C=O groups of GA have reacted with OH groups of the PVA chain [24].

In the infrared spectrum of the PVA/HA 4/1 gel (Figure 4C), the C-OH, C-O-C, and O-C-O vibrations are observed at slightly lower frequencies (1045, 1085 cm^−1^) and have an intensity ratio of 1.11 as compared to 0.86 in the PVA/HA 1/0 sample. A significant broadening and increase in the intensity of the band at 1174 cm^−1^ are also observed. These spectral changes can be rationalized on grounds of a change in the number and distribution of crosslinks in the PVA/HA 4/1 network.

Diffusion of riboflavin into the PVA/HA 4/1 network determines small changes in the ν_C-C_ and ν_C-O_ spectral region (main bands listed in Table S1). The irradiation of the PVA/HA/riboflavin sample determines the decrease in the intensity ratio of the bands at 1048 and 1083 cm^−1^ from 1.05 (prior to irradiation) to 0.89 (after irradiation), a value similar to that in the PVA/HA 1/0 gel (0.86). Irradiation also causes the disappearance of the broad band with a maximum at 1174 cm^−1^ that is characteristic of crosslinked PVA. We believe that these spectral trends collectively indicate that UV-A irradiation in the presence of riboflavin determines a slight decrease in the number of crosslinks. This hypothesis is supported by the rheological data discussed in Section 2.5, specifically by the lower values of the elastic modulus G′ for the PVA/HA/riboflavin/UV system as compared to PVA/HA.

### 2.5. Rheological Behavior of PVA/HA Hydrogels

**Figure 5:**
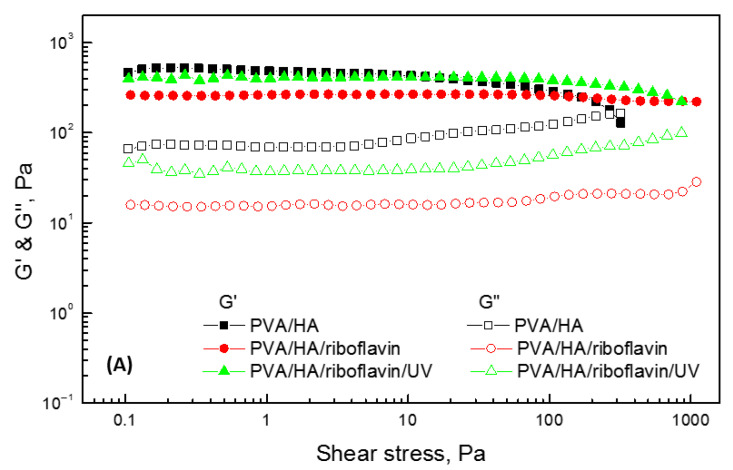

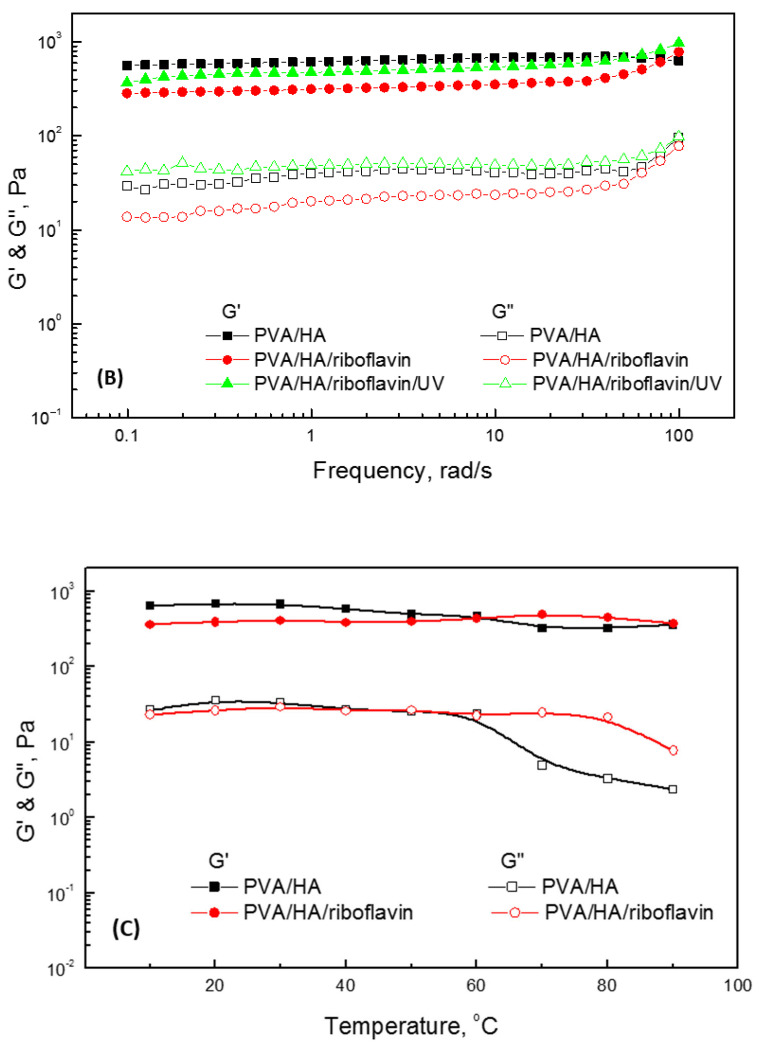
The dependence of the viscoelastic moduli G′ (elastic modulus) and G″ (viscous modulus) on shear stress (**A**), frequency (**B**), and temperature (**C**).

The stability domains of the PVA/HA 4/1 hydrogel in the absence or presence of riboflavin, prior to or after UV-A irradiation, were determined by performing amplitude sweep tests. Figure 5A shows the dependence of the viscoelastic moduli on the shear stress in the interval 0.1–1000 Pa at 25 °C. We observe that, up to a shear stress value of 100 Pa, the hydrogels present an elastic rheological behavior, as indicated by the constant values of the viscoelastic moduli and by the higher value of the elastic or storage modulus (G′) as compared to the viscous or loss modulus (G″). For an applied shear stress above 100 Pa, G′ decreases slowly, and the value of G″ is close to that of G′, indicating the instability/deformation of the hydrogel network. This phenomenon is most evident for the sample in the absence of riboflavin, where a cross point of G′ and G″ is observed.

The rheological behavior of the hydrogel systems was also investigated by oscillatory dynamics. Figure 5B shows the evolution of the G′ and G″ values in a frequency interval from 0.1 to 100 rad/s at 25 °C. As can be observed, in the frequency domain under investigation, the G′ value is always higher than that of G″, and the two moduli do not vary significantly with the frequency. This behavior can be explained by considering that there is not enough time for the reversible crosslinks to dissociate at the applied frequencies, reflecting a strong elastic character. The G′ and G″ values show a slight decrease in the presence of riboflavin: PVA/HA > PVA/HA/riboflavin/UV-A > PVA/HA/riboflavin. An exception is noticed in the case of G″, corresponding to the PVA/HA/riboflavin/UV-A sample.

The more pronounced decrease in viscoelastic moduli observed in the presence of riboflavin could be due to a disruption of the PVA/HA network by hydrogen bonding interactions between the hydroxyl groups of riboflavin and the uncrosslinked functional groups on the polymeric chains. UV-A irradiation causes the photolysis of riboflavin, yielding photoproducts like formylmethylflavin and lumichrome that are devoid of hydroxyl groups [29]. As such, irradiation can aid in restoring the original hydrogen bonding pattern of the PVA/HA network, which explains the recovery, to a great extent, of the rheological behavior. Since the G′ modulus of a hydrogel is directly connected to the density of crosslinks, we consider that the slightly lower G′ value observed for the irradiated sample as compared to the PVA/HA sample can be correlated to the infrared data that indicate a slight decrease in the density of crosslinks for the irradiated sample. This could be an effect of the presence of the HO^•^ radicals detected by EPR. Their ability to degrade PVA [30] and crosslinked HA gels [31] has been previously documented. However, in the case of the PVA/HA 4/1 gel, the rheological data demonstrate that the effect of HO^•^ radicals on the macroscopic properties of the hydrogel is minimal.

The rheological behavior of the PVA/HA and PVA/HA/riboflavin samples as a function of temperature was also analyzed in the temperature range of 10 to 90 °C (Figure 5C). The values of the rheological moduli show a mild decrease with increasing temperature, especially in the case of the PVA/HA hydrogel at temperatures higher than 60 °C. The order of magnitude of G′ is greater than that of G″ in this temperature range. This suggests that the gel phase is maintained even at high temperatures [32]. Moreover, no cross point between G′ and G″ is observed in the dependence of the viscoelastic moduli on the temperature, which indicates the fact that no sol-gel transition occurs.

## 3. Conclusions

To summarize, in this study we investigated the physicochemical properties of hydrogels relevant for ophthalmic applications resulting from IPN generation via crosslinking of PVA and HA with their respective crosslinking agents. The swelling behavior of the gels was characterized as a function of the polymer ratio. The PVA/HA 4/1 volume ratio yielded the optimal balance between the properties of the two polymers, ensuring the highest swelling capacity. Rheological measurements revealed that the hydrogels present an elastic behavior and that the gel phase is stable even at a high temperature of 60 °C.

As a novelty, EPR spectroscopy, providing information at the molecular scale, was used (i) to demonstrate, by spin labeling the HA polymer, the immobilization of the HA network within the IPN structure; (ii) to investigate the diffusion of small paramagnetic molecules across a gel/gel interface, revealing the occurrence of molecular exchange and some degree of self-repair of the hydrogel network; and (iii) to evidence the ROS generated within the gel in the presence of riboflavin under UV-A irradiation. Moreover, visual assessment led us to believe that the PVA/HA 2/3 hydrogel also presents some degree of self-healing (Figure S4), that is, the breaking of the physical network at high stress and its reforming once the stress is removed. This interesting property of PVA/HA hydrogels will be confirmed in future studies by means of rheological measurements.

Corroborating the EPR, infrared spectroscopy, and rheological data, we showed that the formation of HO^•^ radicals induces only slight changes in the global, macroscopic behavior of the hydrogels. This observation can be of use for the future application of these IPN systems as components of artificial tears or as artificial lenses.

## 4. Materials and Methods

### 4.1. General

Polyvinyl alcohol (PVA, 99+% hydrolyzed, average molecular weight 85,000–124,000 g/mol) and hyaluronic acid (HA, sodium salt, 95%, molecular weight 1.5–2.2 million g/mol) were purchased from Sigma-Aldrich (St. Louis, MO, USA) and Acros Organics (Geel, Belgium), respectively. Crosslinkers glutaraldehyde (GA, 25 wt.% in water) and divinyl sulfone (DVS, 97%, stabilized with 0.05% hydroquinone) were acquired from Sigma-Aldrich and Alfa Aesar (Ward Hill, MA, USA), respectively. 1-ethyl-3-(3-dimethylaminopropyl)-carbodiimide hydrochloride (EDC), *N*-hydroxysulfosuccinimide (NHSS), 4-amino-TEMPO, and 5,5-dimethyl-1-pyrroline *N*-oxide (DMPO) were purchased from Sigma-Aldrich. Riboflavin was obtained from Carl Roth (Karlsruhe, Germany).

### 4.2. Preparation of PVA/HA Hydrogel Systems

Stock polymer solutions were prepared as follows. The PVA solution (5 wt.%) was prepared by dissolving PVA in water and stirring for 8 h at 80 °C. The HA solution (1 wt.%) was prepared by dissolving HA in water and stirring for 1 h at room temperature. Crosslinker solutions were freshly prepared prior to each experiment. The GA solution (0.35 vol.%) was prepared by adding 1 mL GA 2.5 vol.% to a mixture of 2 mL methanol 50 vol.%, 3 mL acetic acid 10 vol.%, and 1 mL sulfuric acid 10 vol.% (aqueous solutions). Methanol as a quencher, acetic acid as a pH controller, and sulfuric acid as a catalyst were used in order to accelerate the crosslinking reaction rate [33]. A 16 vol.% DVS aqueous solution was prepared.

To obtain hydrogel systems, the two polymer solutions were mixed in the following PVA/HA volume ratios: 0/1, 1/4, 2/3, 1/1, 3/2, 4/1, and 1/0. Crosslinking agent(s) were added at a PVA/GA and/or DVS/HA molar ratio of 0.35, in accordance with literature data [6]. Thus, in order to prepare the PVA/HA 1/0 sample, 0.28 mL of GA 0.35 vol.% was added to 1 mL of 5 wt.% PVA. To prepare the PVA/HA 0/1 sample, 6 μL of DVS 16 vol.% were added to 1 mL of 1 wt.% HA. The same polymer-to-crosslinker volumetric ratios were used when preparing the other PVA/HA samples. The PVA/HA samples were then stirred for 1 h and stored overnight to allow gel formation. Control samples containing PVA/DVS or HA/GA were prepared in order to ascertain that DVS and GA do not crosslink PVA and HA, respectively, at the molar ratios used in this study (Figure S5). For loading riboflavin into the PVA/HA 4/1 hydrogel, riboflavin (0.1 mg) was dissolved in PVA/HA 4/1 solution (1 mL), and the mixture was allowed to form the gel overnight in the presence of the crosslinking agents. For loading 4-amino-TEMPO, 5 mg of the spin probe were dissolved in 1 mL of PVA/HA 4/1 solution, and the same procedure was applied.

### 4.3. Instrumentation

The EPR spectra were recorded on an X-band spectrometer FA100 from JEOL Resonance Inc. (Tokyo, Japan) equipped with a cylindrical type resonator TE011. For sample irradiation, the UV light emitted by a 500 W mercury arc lamp (LOT-Quantum Design, Darmstadt, Germany) was passed through a UV irradiation accessory (ES-UVAT1, JEOL Resonance Inc., including a 360 nm HOYA colored optical glass filter ES-13020FL) connected to the cavity of the EPR spectrometer via a condenser lens (ES-UVLL/UVLS, JEOL Resonance Inc.). The following operating parameters were used for measurements at room temperature: microwave power 0.998 mW, frequency modulation 100 kHz, modulation amplitude of 1 G, sweep time 240 s, time constant 0.1 s, and magnetic field scan range 100 G. For spin trapping experiments, a sweep time of 60 s was used.

FTIR spectra of PVA/HA dried films were recorded on an iS10 FT-IR spectrometer from Thermo Scientific Nicolet (Waltham, MA, USA).

The viscoelastic behavior of PVA/HA hydrogels was determined using a Kinexus Pro rheometer (Malvern Instruments Ltd., Worcestershire, UK). The temperature was monitored with a compact circulator thermostat CF41 from JULABO GmbH (Seelbach, Germany). Thin hydrogel films (thickness of ~1 mm) were placed between the cone-plate geometries of the rheometer: the fixed plate has a diameter of 55 mm; the plate executing the imposed oscillatory motion has a diameter of 20 mm at an angle of 1°; the gap between plates is 0.7 mm. The linear viscoelastic region (LVR) of the hydrogels was determined by an amplitude sweep test. Dynamic oscillatory tests were also performed, consisting of the stepwise increase in the frequency from 0.1 to 100 rad/s while maintaining constant the deformation (determined from LVR) and the temperature (25.0 ± 0.1 °C). The viscoelastic behavior of the hydrogels was also investigated as a function of temperature (10–100 °C), at constant frequency and deformation.

### 4.4. Swelling Capacity of PVA/HA Hydrogels

The amount of water absorbed by the PVA/HA hydrogels was determined by gravimetric analysis. The hydrogels were dried in ambient conditions to a constant weight (the dry weight, *W_d_*, in g), then immersed in distilled water and weighed at regular time intervals (the hydrated weight, *W_h_*, in g) until equilibrium was reached. The hydrated weight was measured after gently wiping off the excess water from the surface of the gel using filter paper. The water uptake was expressed in terms of the dynamic swelling ratio (*S*) and the equilibrium water content (EWC), calculated as shown in Equations (1) and (2), respectively [34]. Experimental errors from duplicate measurements were within ±6.5% for all samples.

(1) $$S(\%)=\frac{W_{h}-W_{d}}{W_{d}}\times 100$$

(2) $$EWC(\%)=\frac{W_{h}-W_{d}}{W_{h}}\times 100$$

### 4.5. Synthesis of Spin Labeled Hyaluronic Acid

Spin-labeled hyaluronic acid (HA^•^) was prepared by the reaction of HA with 4-amino-TEMPO in the presence of EDC and NHSS to activate the carboxylic acid group of HA. This procedure was described in detail in ref. [35].

### 4.6. Reactive Oxygen Species Generation in PVA/HA Hydrogels

The generation of radical species in PVA/HA hydrogels, in the absence or presence of loaded riboflavin as a photoinitiator, upon UV-A irradiation, was investigated by spin trapping with DMPO (10^−2^ M). The EPR spectra of the DMPO adducts were simulated using the WinSim software version 0.96 [36], applying the LMB1 optimization algorithm [37].

## Figures and Tables

**Table 1:** Water uptake by PVA/HA hydrogels.

PVA/HA	2/3	1/1	3/2	4/1	1/0
Initial water content (%)	96 ± 3	95 ± 3	94 ± 3	95 ± 2	94 ± 4
Equilibrium swelling ratio (%)	175 ± 5	165 ± 5	136 ± 4	258 ± 6	112 ± 5

## Data Availability

The original contributions presented in this study are included in the article/Supplementary Materials. Further inquiries can be directed to the corresponding author.

## Supplementary Materials

The following supporting information can be downloaded at: https://www.mdpi.com/article/10.3390/gels11100773/s1, Figure S1: The PVA/HA systems under investigation after 1 h (A) and after 24 h (B) from mixing the polymer and crosslinker solutions; Figure S2: Swelling behavior of PVA/HA hydrogels in water followed over a longer period of time to ascertain that the maximum water uptake has been reached; Figure S3: The EPR spectra of the spin labeled PVA/HA^•^ 4/1 hydrogel in the absence or in the presence of encapsulated riboflavin, recorded under UV-A irradiation, at different irradiation times; Table S1: Assignments of the main FTIR bands of PVA/HA hydrogels; Figure S4: Two PVA/HA 2/3 hydrogel samples (A) that exhibit self-healing properties upon contact (B,C); Figure S5: Control samples evidencing no gelation of HA in the presence of GA or of PVA in the presence of DVS at the molar ratios used in the study.
