# Supplementary material for: Spectroscopic and Rheological Characterization of Polyvinyl Alcohol/Hyaluronic Acid-Based Systems: Effect of Polymer Ratio and Riboflavin on Hydrogel Properties
Source: Gels. 2025 Sep 25;11(10):773. doi: 10.3390/gels11100773 (PMC12564311; doi:10.3390/gels11100773)
Supplement: Supplementary file 1 [file gels-11-00773-s001.zip › gels-3859739-supplementary.pdf]

Supplementary Material

Spectroscopic and rheological characterization of polyvinyl alcohol/hyaluronic acid-based systems: Effect of polymer ratio and riboflavin on hydrogel properties

Iulia Matei [1], Marius Alexandru Mihai [1], Sorina-Alexandra Leau [1,2], Ludmila Aricov [1], Anca Ruxandra Leonties [1], Elvira Alexandrescu [3] and Gabriela Ionita [1,*]

[1] "Ilie Murgulescu" Institute of Physical Chemistry of the Romanian Academy, 202 Splaiul Independentei, Bucharest 060021, Romania

[2] Department of Analytical Chemistry and Environmental Engineering, Faculty of Chemical Engineering and Biotechnologies, National University of Science and Technology Politehnica of Bucharest, 1-7 Polizu Gheorghe, 011061 Bucharest, Romania

[3] National Institute for Research & Development in Chemistry and Petrochemistry – ICECHIM Bucharest, 202 Splaiul Independentei, Bucharest 060021, Romania

* Corresponding author: ige@icf.ro, gabi2ionita@yahoo.com

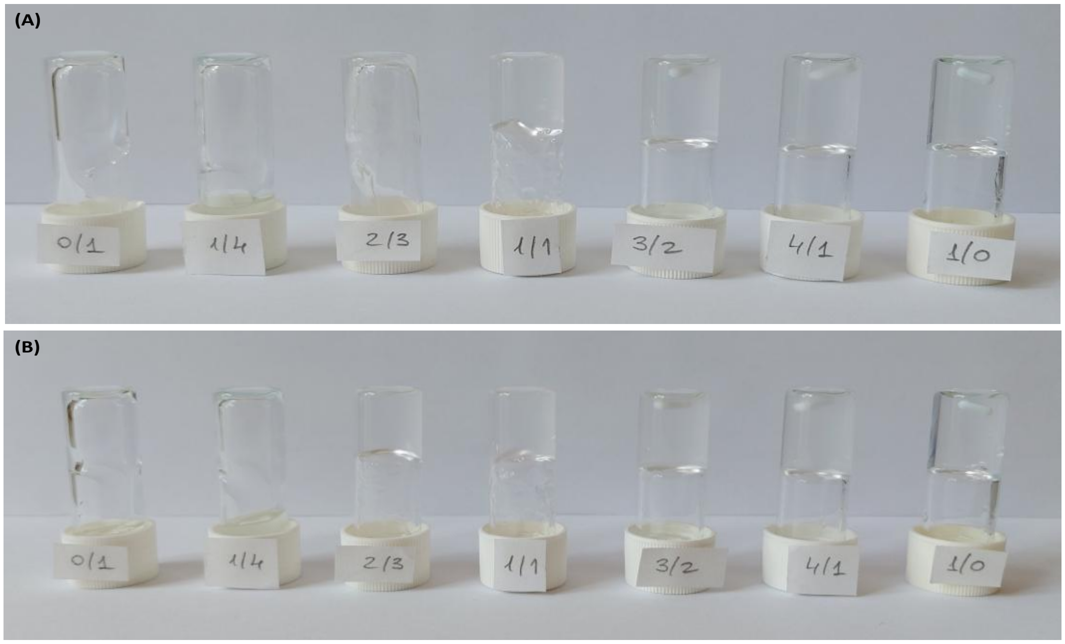

Figure S1. The PVA/HA systems under investigation after 1 h (A) and after 24 h (B) from mixing the polymer and crosslinker solutions.

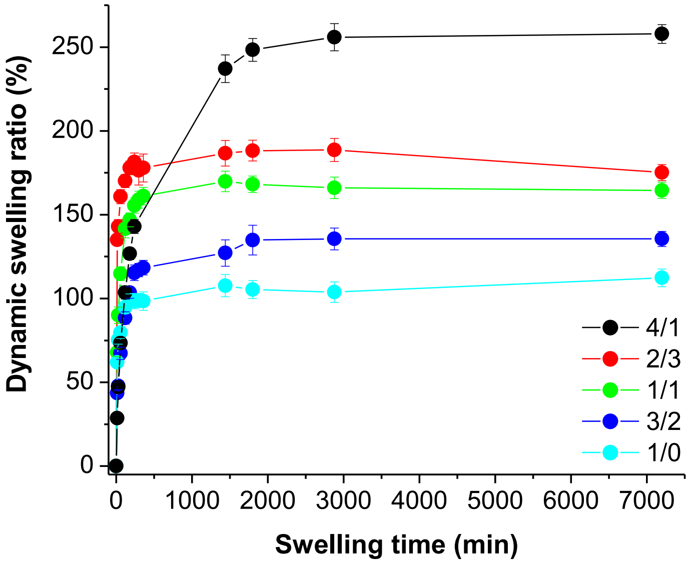

Figure S2. Swelling behavior of PVA/HA hydrogels in water followed over a longer period of time to ascertain that the maximum water uptake has been reached.

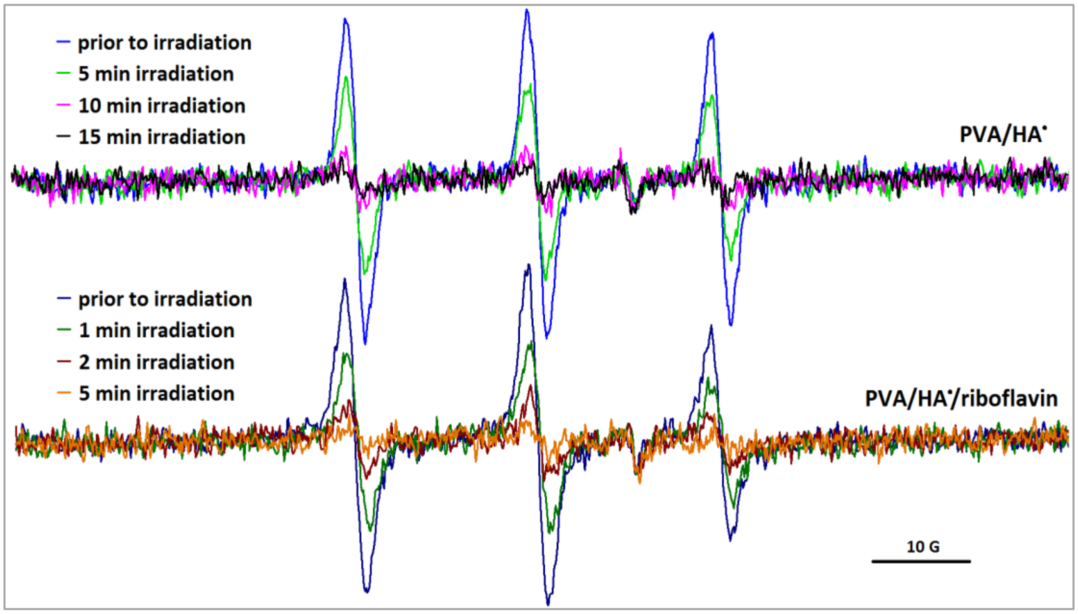

Figure S3. The EPR spectra of the spin-labeled PVA/HA• 4/1 hydrogel in the absence or in the presence of encapsulated riboflavin, recorded under UV-A irradiation, at different irradiation times.

Table S1. Assignments of the main FTIR bands of PVA/HA hydrogels

Sample	Wavenumber (cm^{-1})	Assignment
**HA**	893	saccharide ring deformation
	946	out-of-phase C-H wagging in pyranose ring
	1035 (s), 1077 (sh)	C-OH stretching
	1148	C-O-C stretching
	1319, 1376	amide III bands
	1408	CH_2 bending
	1560 (sh)	amide II band (N-H bending, C-N stretching)
	1605 (s)	C=O stretching in amide (amide I band) and in carboxylate
	2830-2950	symmetric, asymmetric C-H stretching
	3100 (sh)	N-H stretching of N-acetyl side chain
	3130-3600 (s)	O-H stretching, hydrogen bonded
**PVA/HA 0/1** (HA crosslinked with DVS)	895	saccharide ring deformation
	946	out-of-phase C-H wagging in pyranose ring
	1031 (s), 1077 (sh)	C-OH stretching
	**1146**	**C-O-C stretching**
	**1136**	**S=O symmetric stretching**
	1322, 1376	amide III bands
	1409	CH_2 bending
	1560 (sh)	amide II band (N-H bending, C-N stretching)
	1602 (s)	C=O stretching in amide (amide I band) and in carboxylate
	2830-2950	symmetric, asymmetric C-H stretching
	3100 (sh)	N-H stretching of N-acetyl side chain
	3130-3600 (s)	O-H stretching, hydrogen bonded
**PVA**	836	C-O deformation
	1088 (s)	O-H bending coupled with C-OH stretching
	1142 (s)	C-C and C-O stretching
	1236	CH_2 wagging
	1324, 1374 (w)	$C\text{-}CH_3$ deformations
	1417	C-H deformations in CH_2
	1658 (w)	C=O stretching of residual acetate groups*
	2838 (sh), 2908, 2938	symmetric, asymmetric C-H stretching
	3000-3600 (s)	O-H stretching, hydrogen bonded
**PVA/HA 1/0** (PVA crosslinked with GA)	831	C-O deformation
	**1048, 1088 (s)**	O-H bending coupled with C-OH, C-O-C, O-C-O stretching
	1142 (s), **1173, 1201 (w)**	C-C and C-O stretching
	1233	CH_2 wagging
	1328, 1377 (w)	$C\text{-}CH_3$ deformations
	1416	C-H deformations in CH_2
	1653	C=O stretching of residual acetate groups*
	1706 (w)	C=O stretching, unreacted end of GA
	2838 (sh), 2908, 2938	symmetric, asymmetric C-H stretching
	3000-3600	O-H stretching, hydrogen bonded

**PVA/HA 4/1**	838<br>**1045 (s), 1085 (s)**<br>1142, **1174 (s)**<br>1327, 1379 (w)<br>1420<br>1649<br>1691<br>2855 (sh), 2911, 2941<br>3100-3600	C-O deformation (PVA)<br>O-H bending, C-OH, C-O-C, O-C-O stretching (PVA)<br>C-C, C-O stretching (PVA, HA)<br>amide III bands (HA), C-CH_3 deformations (PVA)<br>C-H deformations in CH_2 (PVA)<br>C=O stretching of residual acetate groups* (PVA)<br>C=O stretching, unreacted end of GA<br>symmetric, asymmetric C-H stretching (PVA. HA)<br>O-H stretching, hydrogen bonded (PVA, HA)
**PVA/HA 4/1 + riboflavin**	837<br>1045 (s), 1085 (s)<br>1142, **1183**<br>1327, 1379 (w)<br>1416<br>1649<br>1703<br>2855 (sh), 2911, 2941<br>3100-3600	C-O deformation (PVA)<br>O-H bending, C-OH, C-O-C, O-C-O stretching (PVA)<br>C-C, C-O stretching (PVA, HA)<br>amide III bands (HA), C-CH_3 deformations (PVA)<br>C-H deformations in CH_2 (PVA)<br>C=O stretching of residual acetate groups* (PVA)<br>C=O stretching, unreacted end of GA<br>symmetric, asymmetric C-H stretching (PVA, HA)<br>O-H stretching, hydrogen bonded (PVA, HA)
**PVA/HA 4/1 + riboflavin + UV**	832<br>**1048 (sh), 1083 (s)**<br>1142<br>1235<br>1324, 1376 (w)<br>1417<br>1648<br>1718<br>2848 (sh), 2910, 2938<br>3100-3600	C-O deformation (PVA)<br>O-H bending, C-OH, C-O-C, O-C-O stretching (PVA)<br>C-C, C-O stretching (PVA, HA)<br>CH_2 wagging (PVA)<br>amide III bands (HA), C-CH_3 deformations (PVA)<br>C-H deformations in CH_2 (PVA)<br>C=O stretching of residual acetate groups* (PVA)<br>C=O stretching, unreacted end of GA<br>symmetric, asymmetric C-H stretching (PVA, HA)<br>O-H stretching, hydrogen bonded (PVA, HA)

* Band of vinyl acetate groups persisting after the hydrolysis of polyvinyl acetate to obtain PVA; these groups can also originate in oxidations during manufacturing and/or processing.

Abbreviations: s – strong, w – weak, sh – shoulder

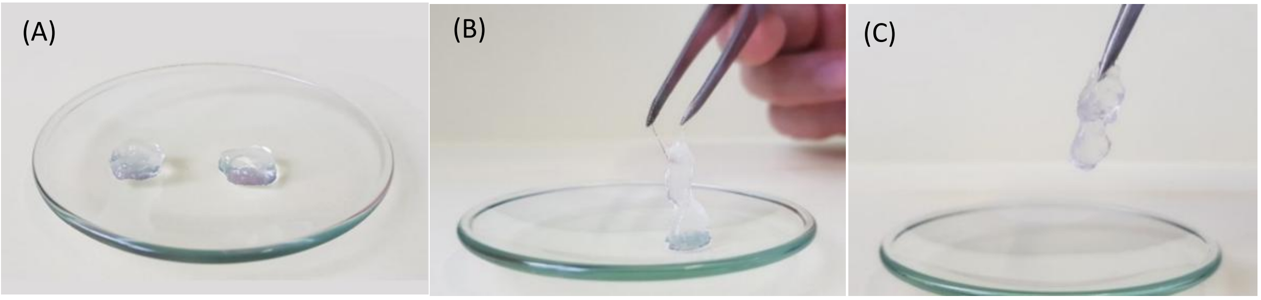

Figure S4. Two PVA/HA 2/3 hydrogel samples (A) that exhibit self-healing properties upon contact (B, C).

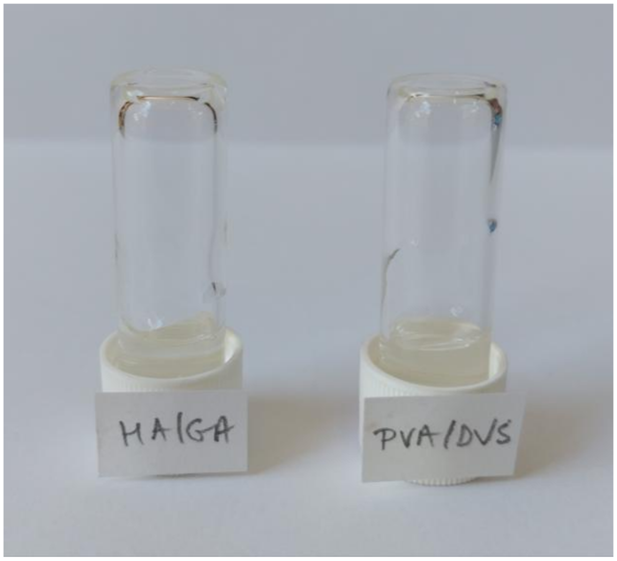

Figure S5. Control samples evidencing no gelation of HA in the presence of GA or of PVA in the presence of DVS at the molar ratios used in the study.
